# Focus on global public health, screening for tuberculosis using colour-coded electronic health signs similar to that employed for screening coronavirus disease 2019 in China

**DOI:** 10.1186/s12967-023-03975-1

**Published:** 2023-02-10

**Authors:** Jian Xu

**Affiliations:** Department of Infectious Disease, The People’s Hospital of Yubei District of Chongqing City, Yubei District, No.23, North Central Park Road, Chongqing, 401120 China

Dear editor,

Communicable diseases remain a major global public health challenge. The ongoing coronavirus disease 2019 (COVID-19) pandemic has placed global attention on the infectious diseases through airborne spread. Among these, tuberculosis (TB) is the most common communicable disease in China [1]. Great efforts have been made to eliminate the public health hazards caused by TB worldwide. In 2015, the World Health Organization (WHO) proposed a strategy to end the TB epidemic by 2035 [2]. However, WHO Global Tuberculosis Report of 2022 reports that there still approximately 10.60 million newly diagnosed TB patients worldwide [3].

China adopted the WHO-recommended directly observed treatment strategy (DOTS) in the 1990s, and the DOTS was implemented in the entire country by 2005 [4]. However, China is still one of the countries with the highest TB burden. The estimated number of new TB cases in China is 780,000 in 2021; The incidence of TB is decreasing annually, from 71.2/100,000 in 2010 to 55/100,000 in 2021 [3], and is expected to drop to 35.9/100,000 by 2030 [5]. However, there are many obstacles to fulfilling the WHO's target. Firstly, a well-designed TB screening strategy involving the government, medical institutions and society is difficult to realize. In addition, Chinese people generally lack a sound awareness of disease prevention and control, and are unwilling to take the initiative to screen for TB. As a result, the detection and diagnosis of TB is relatively insufficient in China. TB-infected individuals and individuals with latent TB hidden in the community pose a risk to healthy persons, resulting in community or family transmission. Therefore, the number of TB may be underestimated in China. Finding a universal TB screening strategy is a top priority for TB control in China.

Since the end of 2019, COVID-19 has caused profound economic and social disruption worldwide, and countries have adopted different prevention and control strategies. Among them, a screening model using colour-coded electronic health sign has been applied in China. A green electronic health sign was assigned to every person if the nucleic-acid test for COVID-19 showed a negative result. If a citizen does not undergo a nucleic acid test within a certain period of time, the colour assigned to the electronic health sign changes (usually yellow), and he or she is not allowed to enter public places. The confirmed COVID-19 patient was given to a red electronic health sign. If a person is in close contact with someone who has COVID-19, he or she will also get a red health sign, advising them to be screened for COVID-19 within a set time.

COVID-19 has aroused interest in communicable diseases and highlighted the importance of asymptomatic transmission. It is unfortunate that the TB has not received similar attention over past decades. Now is an exciting time for national surveillance of TB. Like COVID-19, TB is primary transmitted through the respiratory tract. If actively infected TB patients and those who are still in the incubation phase remain undetected, they could become latent infectious agents of TB, thereby exacerbating the spread of TB. Timely and accurate screening of patients with active TB and those in the incubation period has become an urgent challenge. In this context, our team recommend a whole-population TB screening program and colour-coded electronic health signs for screening TB, similar to the one used for COVID-19 screening in China. In particular, TB related tests, chest X-ray combined with tuberculin test of pure protein derivative (PPD), were applied to screen for TB among general citizen. Yellow and red electronic health sign was assigned to the suspected and confirmed TB cases, respectively. Further check needed among these persons with yellow electronic health sign in order to make clear whether there is any active TB. Antituberculosis treatment should be given to these persons with red electronic health sign. After treatment and recovery, a green electronic health sign was given to them. Intensive screening of close contacts with confirmed individuals is required.

The use of colour-coded electronic health signs to screen for TB, although certain scientific or ethical controversy exist, is still a valuable strategy. Asymptomatic cases who were not detected as part of this TB screening algorithm may have contributed to the spread of TB. The TB community can draw on the comprehensive approaches used to manage COVID-19 to help to achieve the goal of ending TB by 2035. As shown in Fig. 1, TB screening should be conducted based on China’s strategy for screening COVID-19. It is only through multi-sectoral collaborations including government, society and medical institutions that address purpose of ending TB by 2035. The specific screening roadmap are shown in Fig. 2.

**Fig. 1:**
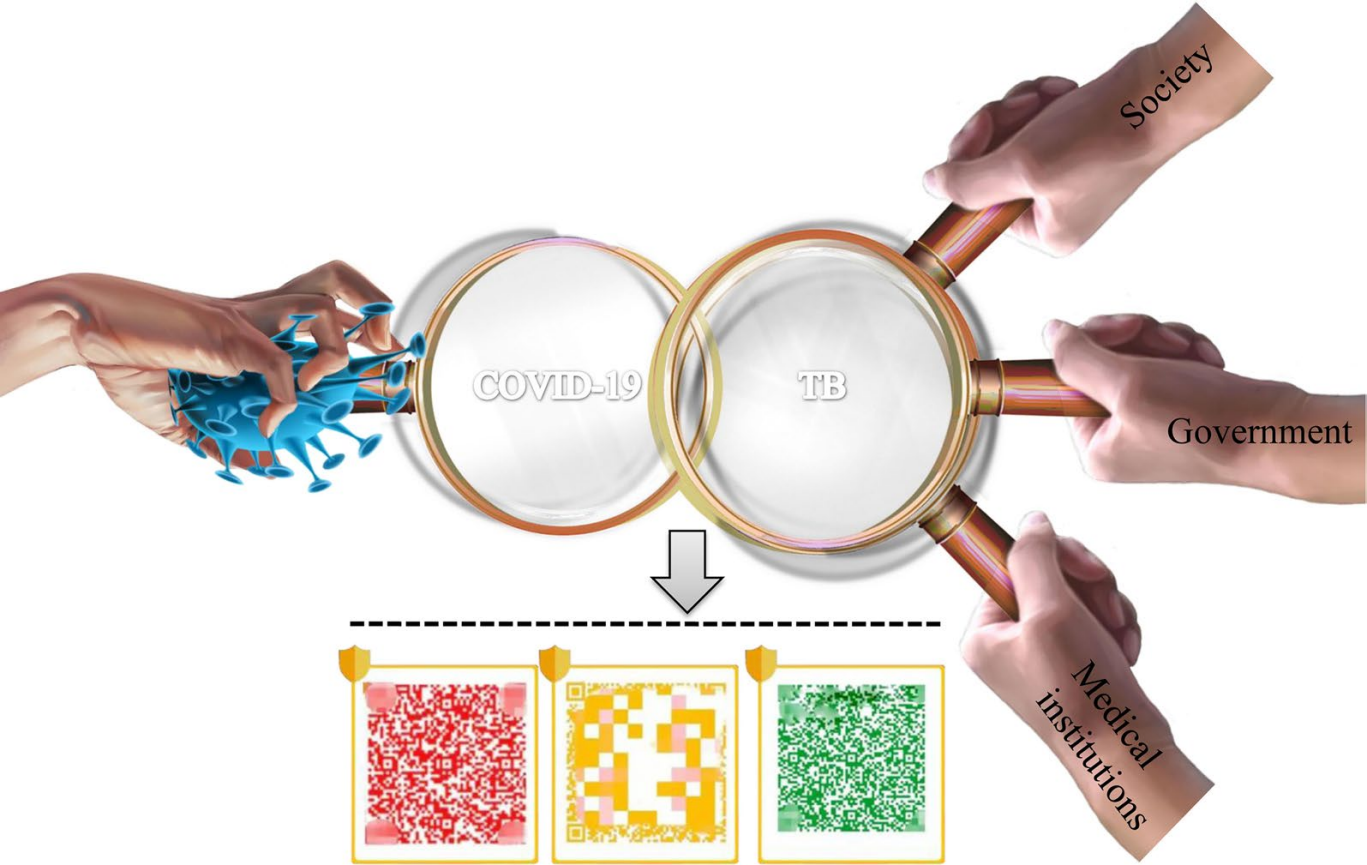
Diagram of tuberculosis screening based on COVID-19 screening method in China

**Fig. 2:**
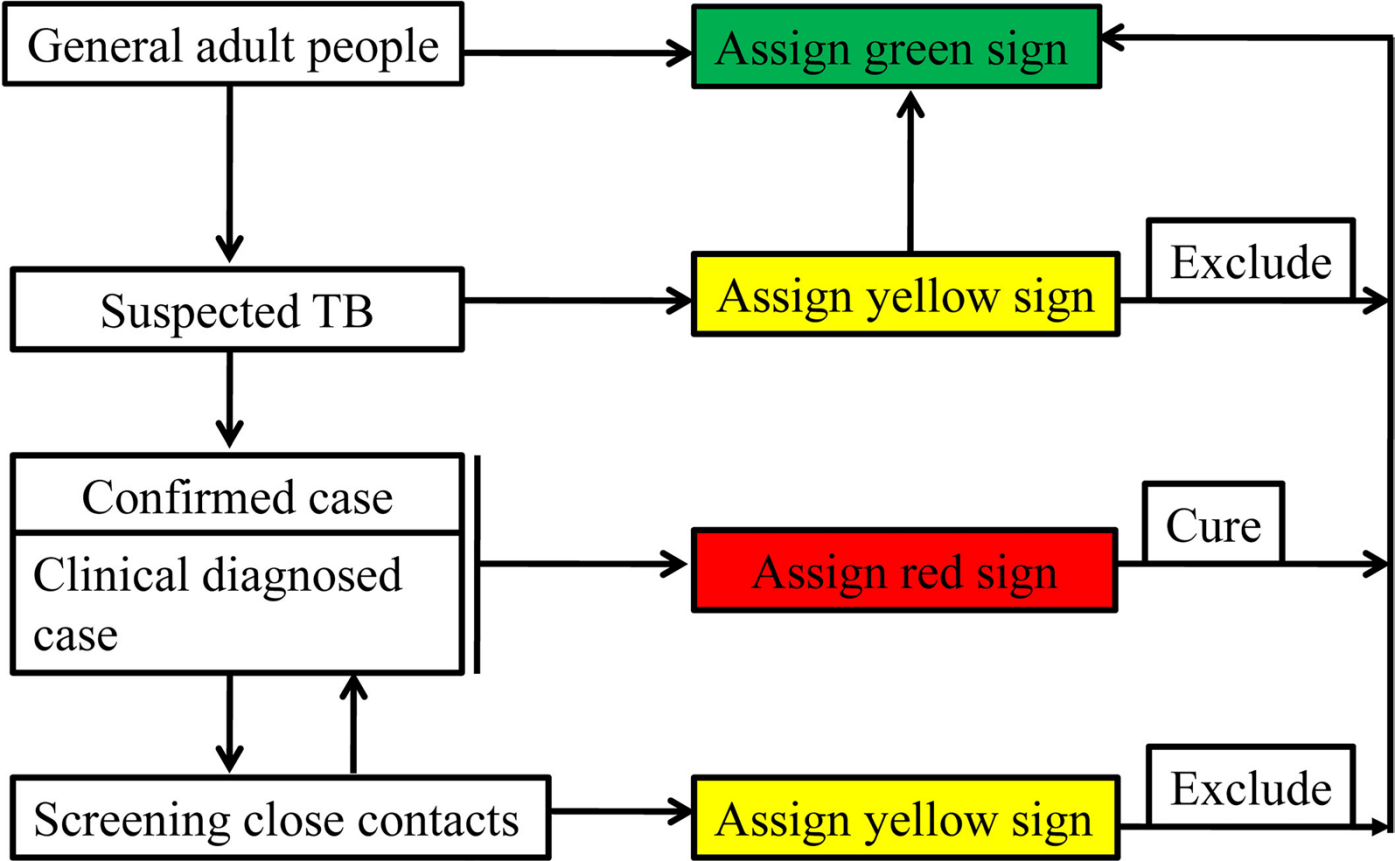
Roadmap of screening TB in China

## Data Availability

Not applicable.
